# Gallbladder Perforation in a Pregnant Patient: A Case Report and Considerations of Surgical Approach

**DOI:** 10.7759/cureus.73679

**Published:** 2024-11-14

**Authors:** Yesika Alejandra Guerra-Juarez, Judith N Mendez-Martinez, Luis Adrian Alvarez-Lozada, Alejandro Quiroga-Garza, Guillermo Jacobo-Baca, Rodrigo E Elizondo-Omaña

**Affiliations:** 1 Human Anatomy Department, Clinical-Surgical Research Group (GICQx), Universidad Autónoma de Nuevo León, Monterrey, MEX; 2 General Surgery Department, Instituto Mexicano del Seguro Social, Monterrey, MEX

**Keywords:** cholecystectomy laparoscopic, gallbladder perforation, minimally invasive surgical procedures, pregnancy, subtotal laparoscopic cholecystectomy

## Abstract

Gallbladder disease is a frequent indication for non-obstetric surgical intervention during pregnancy. Gallbladder perforation (GBP) during pregnancy is an uncommon but severe pathology that usually requires immediate attention, and it represents a challenge for surgeons. We present the case of a GBP in a pregnant patient alongside a discussion of available surgical approaches.

A 32-year-old pregnant patient at 21.5 weeks of gestation presented with a four-day history of abdominal pain. Two weeks prior, she underwent an endoscopic retrograde cholangiopancreatography (ERCP) for stone removal and biliary and pancreatic prostheses placement due to choledocholithiasis. The patient was admitted for a follow-up ERCP with lithotripsy. A laparoscopic total cholecystectomy was indicated, during which abundant purulent secretion, four stones in the abdominal cavity, and the transverse colon in close contact with the gallbladder were identified. A critical view of safety was obtained, and type 2B subtotal cholecystectomy was performed, with abscess drainage and Blake drainage placed. Postoperative follow-up and gestation were uneventful.

Although uncommon, GBP in pregnancy should always be considered in patients with a history of gallbladder symptomatology. An early diagnosis allows for an opportune surgical approach, which should not be delayed. This allows for the best outcomes in pregnancy for both the fetus and the gestational parent.

## Introduction

Cholecystolithiasis affects approximately 10%-20% of the general population, with a 2-4 times higher prevalence in women and 20% of these being under the age of 40. Gallbladder disease is the leading non-obstetric cause of the most common hospitalizations in the first year after childbirth and the second most frequent indication for non-obstetric surgical interventions during pregnancy [1].

Gallbladder perforation (GBP), while uncommon, poses a significant risk to life in cases of acute cholecystitis, with mortality rates ranging from 12% to 42% according to reported data [2]. In 1934, Niemeier classified this complication into three types that are still recognized today as type 1 (chronic perforation with cholecystic-enteric fistula), type 2 (perforation with abscess), and type 3 (free perforation) [3]. Despite 90 years of its classification, authors continue to debate the optimal management approach, technique, and time of intervention [4,5].

In the Society of American Gastrointestinal and Endoscopic Surgeons guidelines, it has been described that during pregnancy, the therapeutic approach may vary depending on each patient's condition. However, better outcomes are reported in performing endoscopic retrograde cholangiopancreatography (ERCP) followed by laparoscopic cholecystectomy, compared to laparotomy. One of the main benefits is a lower incidence of spontaneous abortions and preterm births. Additionally, no difference has been reported in post-surgical complications according to the trimester of pregnancy. We present the case of a GBP in a patient with a 21.5-week gestation treated promptly with ERCP and laparoscopic cholecystectomy. The current case report follows the Surgical Case Report checklist for documenting clinical cases.

## Case presentation

A 32-year-old pregnant patient presents in the obstetrics emergency department referred by the gastroenterology service due to a four-day history of ongoing abdominal pain. Two weeks prior, the patient was diagnosed with choledocholithiasis and treated with biliary and pancreatic prostheses, and retrograde cholangiopancreatography (ERCP), during which one and a half stones were removed. Upon physical examination, the patient presented normal vital signs, the uterine fundus measured 19 cm, and the fetal heart rate (FHR) was 113 bpm. Vaginal examination revealed a free soft closed cervix, without palpable uterine activity, and intact membranes. The abdomen was distended due to pregnancy and painful upon palpation in the epigastrium and the right hypochondrium, although normal peristalsis was noted. A positive Murphy sign was observed. Blood laboratory tests are reported in Table 1.

**Table 1:** Blood laboratory tests Values are based on the American College of Clinical Pharmacy. RCB: red blood cell count, HGB: hemoglobin, HCT: hematocrit, WBC: white blood count, NEU: neutrophils, PLT: platelet count, GLU: glucose, ALP: alkaline phosphatase, ALT: alanine aminotransferase, AST: aspartate aminotransferase, TB: total bilirubin, DB: direct bilirubin, UCB: unconjugated bilirubin, CRP: C-reactive protein, PT: prothrombin time, PTT: partial thromboplastin time, Fg: fibrinogen

Blood test	Value	Reference ranges
RCB	3.42 × 10^6^ cells/mm^3^	4.5-5.9 × 10^6^ cells/mm^3^ (men), 4.1-5.1 × 10^6^ cells/mm^3^ (women)
HGB	10.10 g/dL	14-18 g/dL (men), 12-16 g/dL (women)
HCT	30.9%	42%-50% (men), 36%-45% (women)
WBC	17.4 × 10^3^ cells/mm^3^	4.5-11.0 × 10^3^ cells/mm^3^
NEU	15.6/mm^3^	2500-8000/mm^3^
PLT	217 cells/mm^3^	150,000-350,000 cells/mm^3^
GLU	65 mg/dL	70-110 mg/dL
ALP	148 IU/L	30-120 IU/L (adults), 150-420 IU/L (children)
ALT	33 U/L	10-40 U/L
AST	29 U/L	10-30 U/L
TB	1.1 mg/dL	0.3-1.2 mg/dL
DB	0.4 mg/dL	0.1-0.3 mg/dL
UCB	0.7 mg/dL	0.2-0.8 mg/dL
CRP	36 mg/L	0.08-3.1 mg/L
PT	15.4 seconds	10-13 seconds
PTT	30.3 seconds	25-40 seconds
Fg	>700 mg%	150-400 mg%

**Figure 1:**
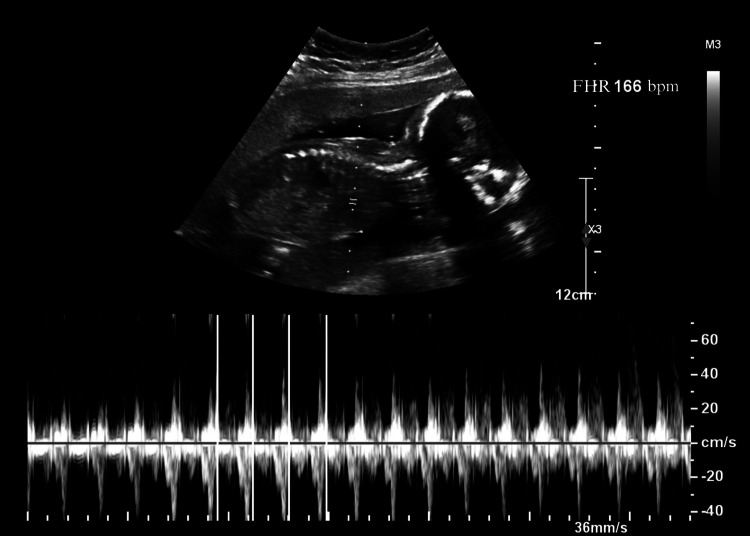
Obstetric ultrasonography Fetus in breech presentation with an FHR of 166 bpm. FHR: fetal heart rate

**Figure 2:**
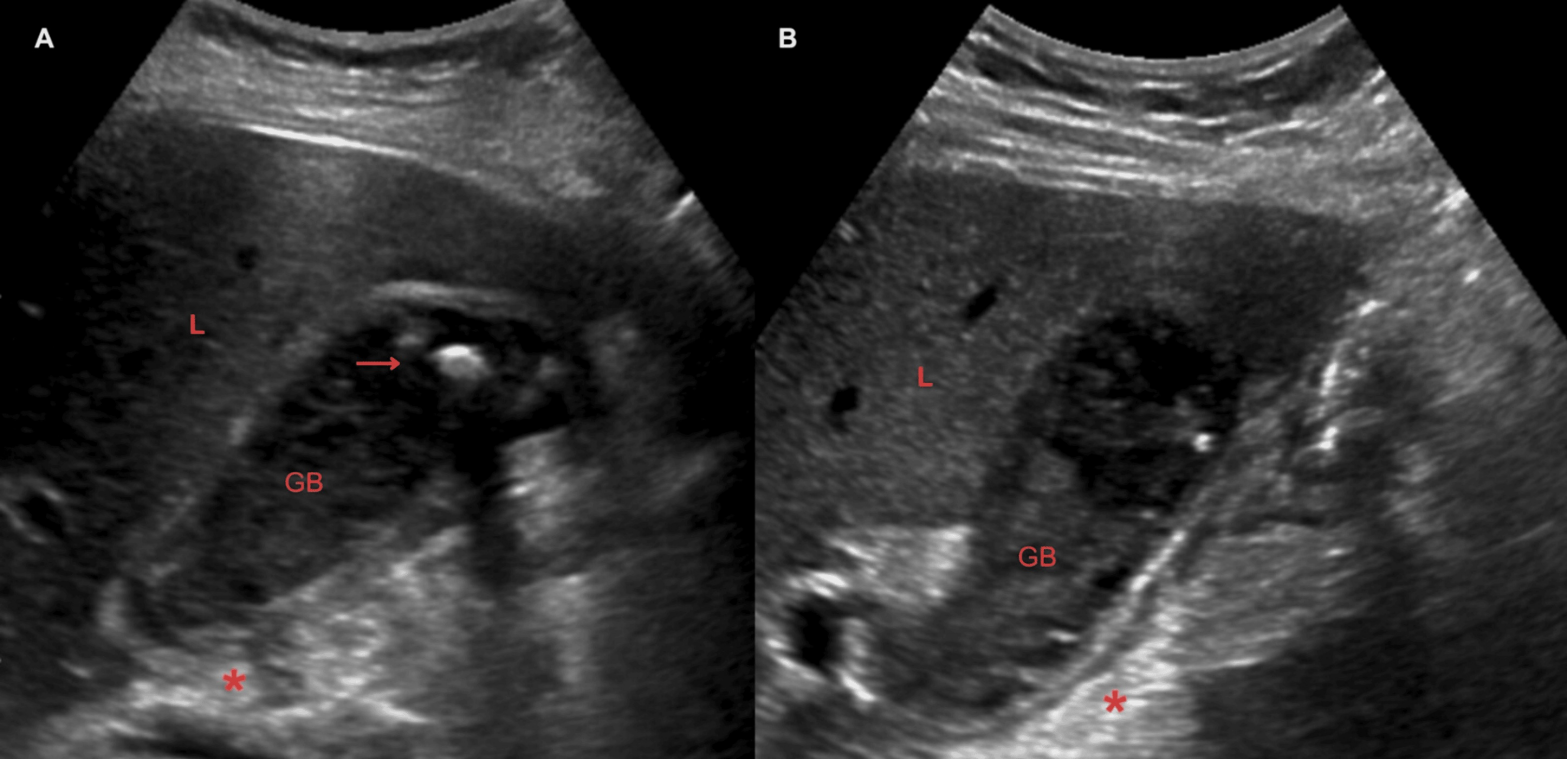
Abdominal ultrasound The arrow shows a rounded echogenic image. The asterisk (*) shows edema and perivesicular fluid. A: GB with lithiasis. B: GB and Hartmann pouch. GB: gallbladder, L: liver

Obstetric ultrasonography (Figure 1) showed a fetus in breech presentation without pathological alterations. Abdominal ultrasound (Figure 2) reported gallbladder with multiple rounded echogenic images in relation to lithiasis and thick biliary sludge. Edema and perivesicular fluid are identified. A dilated common bile duct with a rounded echogenic image suggestive of lithiasis is identified. Radiologist diagnosis impression reported data suggestive of cholecystitis and cholecystocholedocholithiasis. The patient was hospitalized for treatment with a double antibiotics regimen and nonsteroidal anti-inflammatory drugs (NSAIDs).

The next day, an ERCP was performed under general anesthesia in the gastroenterology department. Removal of the biliary prosthesis was performed using a snare, without complications. The cholangiogram showed a common bile duct of 8.5 mm with multiple filling defects (stones of 6.4 mm, 8 mm, 7.1 mm, and 4.5 mm). Balloon sweeps were performed, showing drainage of bile sludge and pus. The basket was advanced, and lithotripsy was successfully performed. Postoperatively, general surgery was consulted, and a laparoscopic total cholecystectomy was scheduled for the next day.

Transoperative findings included the transverse colon in close contact with the gallbladder. Dissection by planes revealed abundant purulent secretion, a gallbladder with a friable and necrotic wall, and four stones in the abdominal cavity, which were collected. No plane of separation between the colon and gallbladder was identified. Type 2B subtotal cholecystectomy (Purzner's classification) is performed, accompanied by drainage of pyogenic abscess in the gallbladder and placement of Blake-type drainage. Hemostasis was confirmed, and the procedure was finished. The patient was discharged on the second postoperative day, and Blake drainage was removed on the seventh postoperative day due to scarce seromatic drainage. Follow-up was uneventful until the birth of the pregnancy.

## Discussion

Pregnancy increases the risk of biliary disease due to physiological changes that promote the formation of gallstones (lithogenesis). Approximately one in every 500 pregnant patients will require non-obstetric abdominal surgery during this period, with acute appendicitis and cholecystitis being the most common causes [6]. Cholecystitis during pregnancy mainly occurs in the last trimester, primarily because pregnancy itself is considered a risk factor for the formation of biliary sludge. While some researchers consider the frequency of biliary sludge to be similar between nulliparous and multiparous patients, others have reported the number of pregnancies increases the risk of developing gallstones, with most occurring in the second and third trimesters [7].

Perforations in the biliary tract are extremely rare and sparsely documented in the literature. However, this condition can be caused by various factors, such as increased pressure within the ducts due to gallstones, thrombosis of a blood vessel in the wall, infection in the biliary duct, and perforation caused by stones at a point of obstruction [4,8]. Although uncommon, any symptom presented by patients with a history of biliary problems should be investigated promptly to avoid serious and potentially life-threatening complications [9,10]. Cases of acute cholecystitis during pregnancy that do not respond to medical management require surgical consultation to assess the need for surgical intervention. The approach can be either open cholecystectomy (OC) or laparoscopic cholecystectomy (LC). A systematic review and meta-analysis comparing these methods reported LC as a safer and preferable option for both the pregnant patients and the fetus, with considerably fewer complications and shorter hospitalization periods, without significant differences in operating times [6,11]. This advantage for LC is particularly evident during the first and second trimesters due to the technical limitations such as poor vision, difficulty in port placement, and limited space caused by the large gravid uterus during the third trimester. Despite these advantages, many surgeons remain reluctant and opt for an OC approach for cholecystectomy in the early stages of pregnancy. However, LC continues to gain popularity as more surgeons train in this technique and as it becomes more widely available [11].

While the therapeutic approach to biliary disease during pregnancy requires choosing the approach with the greatest benefit for both the mother and the fetus, within another systematic review, LC had shorter operation times and faster recovery and mobilization. This results in reduced fetal exposure to anesthesia and analgesic medications [12]. In the same manner, it is documented that maternal complications such as cesarean section, hysterectomy, maternal dehydration, or preeclampsia were found in 334 out of 9413 (3.5%) patients undergoing LC and 100 out of 1219 (8.2%) patients in the OC group. Among these patients, it was reported that 901 out of 9413 (9.6%) patients in the LC group and 211 out of 1219 (17.3%) in the OC group experienced surgical complications such as bile duct injuries and injuries to other organs or hollow viscera. Fetal complications were also assessed, including fetal loss, fetal distress, threatened preterm labor, and preterm birth, affecting 346 out of 8807 (3.9%) patients undergoing LC and 139 out of 1161 (12%) patients in the OC group [11].

Some specialists consider a conservative treatment more viable to avoid risking the life of the mother or the fetus. However, according to research, patients with symptomatic cholelithiasis treated with a conservative approach experienced more episodes of recurrent biliary symptoms and had a higher number of emergency department visits and hospitalizations related to biliary complications. It is also associated with early induction of labor (14%) and increased cesarean section rate (35%) [13].

Based on the review of the literature shown in Table 2, the approach taken with our patient was appropriate as optimal conditions existed for performing surgical treatment, even during a difficult cholecystectomy due to the presence of GBP [10,14]. However, data is limited to isolated case reports. Cohorts, case series, and revisions are needed to compare the outcomes and safety of surgical approach technique for GBP in gestating women.

**Table 2:** Literature review WOG: weeks of gestation, OC: open cholecystectomy, LC: laparoscopic cholecystectomy

Study	Age	WOG	Diagnosis	Clinical data	Therapeutic approach	Postoperative
Talwar et al. (2006), India [8]	28	30	Cholelithiasis and gallbladder perforation	Peritonitis due to intestinal perforation	LC with peritoneal lavage	Uneventful
Petrozza et al. (1995), United States [10]	28	29	Cholelithiasis and gallbladder perforation	Peritonitis, polysubstance abuser, hepatitis C (+), metabolic acidosis, sepsis	LC	Complicated by worsening sepsis and acidosis
Mosawi et al. (2019), Afghanistan [14]	17	22	Gallbladder perforation and cholecystitis	*Ascaris lumbricoides* in the gallbladder and common bile duct	OC	None reported

## Conclusions

A gestating woman may safely undergo LC if biliary colic symptoms persist or escalate despite dietary modifications and medical management. Delays in surgical management may lead to complications, but further research is needed specifically for GBP. The choice of approach is a combination of hospital resources, surgeon expertise and skills, and patient decision.
